# Social isolation, coping efficacy, and social well-being over time in patients with lung cancer

**DOI:** 10.1007/s10865-024-00508-z

**Published:** 2024-07-29

**Authors:** Victoria J. Dunsmore, Shevaun D. Neupert

**Affiliations:** 1 Department of Psychology, North Carolina State University, Raleigh, USA; 2 UNC Chapel Hill, Lineberger Comprehensive Cancer Center, Chapel Hill, USA

**Keywords:** Coping Efficacy, Social Isolation, Social Well-Being, Lung Cancer

## Abstract

**Background:**

Little work has examined how coping efficacy and lung cancer-related social isolation relate to social well-being in the context of a patient’s computed tomography (CT) scan. Researchers tested the cross-sectional relationship of social isolation and social well-being, and the longitudinal relationship between coping efficacy and social well-being before CT scans.

**Method:**

25 patients with lung cancer, within 6 months of their upcoming CT scan, participated. Baseline surveys collected clinical information, demographics, and social isolation; repeated monthly surveys collected coping efficacy and social well-being every 30 days until one’s scan.

**Results:**

[Cross-sectional] High levels of social isolation were associated with low levels of social well-being.

[Longitudinal] On months patients reported high coping efficacy, they also reported increases in social well-being.

**Conclusions:**

Social interventions may improve well-being among those with lung cancer as our work shows that getting and receiving support are strongly related to well-being in the time surrounding a scan.

**Supplementary Information:**

The online version contains supplementary material available at 10.1007/s10865-024-00508-z.

## Introduction

Lung Cancer is the leading cause of cancer deaths and is the 2nd most diagnosed cancer in the United States (ACS, 2019). Regardless of prognosis, these patients are expected to undergo repeated computed tomography (CT) scans to screen progression in frequency ranging from every few months to annually (ACS, 2022). These scans can elicit increased levels of anxiety about their potential results in the months and days prior to one’s scan (Dunsmore & Neupert, 2023) and may also serve as a reminder of the stigma the patient has experienced or continues to experience, because of their diagnosis. Lung cancer-related stigma is the result of the public perception that behavioral ‘choices’, including smoking, caused their illness (Bayer et al., 2006; Bell et al., 2010; Gritz et al., 2007), and can include experiences of social isolation, shame and blame, and discrimination; all of which are typically considered to be stable (Carter-Harris et al., 2014; Holt-Lunstad et al., 2022). People diagnosed with lung cancer are at a greater risk of experiencing social isolation, a contributing component of stigma, due to the external and internalized experiences of stigma (Gonzalez et al., 2012; Hamann et al., 2021; Hathaway et al., 2022; Polański et al., 2022; Rigney et al., 2021; Takemura et al., 2022; Webb et al., 2019; Williamson et al., 2020). These factors significantly impact one’s quality of life; in particular, social well-being, which is characterized as receiving social and/or familial support (Brown Johnson et al., 2014; Chambers et al., 2012; Cella et al., 1995)

Taking this into account, it is important to understand one’s confidence in coping skills in the context of repeated scans, namely coping efficacy which stems from Bandura’s (1997) self-efficacy theory in that it points to one’s confidence in their ability to perform the necessary actions to achieve a desired outcome. A scoping review recently noted that social relationships impact how patients with cancer cope with the commonly experienced phenomenon ‘scanxiety’, that is associated with repeated scans (Derry-Vick et al., 2023). Another study found that patients who reported higher social isolation had higher distress on their imaging day compared to those who had strong social connections (Morreale et a., 2020). Coping efficacy has also been shown to fluctuate across time and be related to receiving social support among caregivers of patients with cancer, such that on days caregivers received social support they reported reduced negative affect even if they had low coping efficacy (Kroemeke et al., 2021). Coping efficacy has also been shown to predict quality of life among patients with resected lung cancer 3 months after surgery (Chen et al., 2018; Chirico et al., 2017).

To examine how appraisal of social isolation and coping impact social well-being in the time surrounding a specific stressful cancer-related event (CT scans), researchers employed both the transactional model of stress and coping (Lazarus et al., 1984) and the Conceptual Model of Lung Cancer Stigma (Hamann et al., 2014). Namely, researchers focused on (1) social isolation as a proxy of perceived/felt stigma based on the devaluation of others, and (2) personal attributes of coping, specifically coping efficacy. The current study applies these models to look at social well-being among patients who are receiving a repeated scan. By focusing on social isolation, and coping efficacy, future work may be able to disrupt the internalization of stigma through social isolation, which can result in persistent maladaptive consequences as defined by Hamann et al. (2014).

## Future-oriented coping and anxiety with scans

Future-oriented coping involves imagining oneself in various hypothetical states to prepare for upcoming events and has been shown to be advantageous in promoting positive outcomes (Aspinwall et al., 1997; Szpunar et al., 2014). Proactive coping is a form of future-oriented coping where individuals engage in behaviors to modify how a stressor unfolds (Aspinwall et al., 1997). It has been investigated as both a buffer for health-related stigma (Chaudoir et al., 2012), and a tool for health-related self-efficacy (Tielemans et al., 2015). In the present study, researchers incorporate proactive coping as a covariate, as it may modify one’s quality of life outcomes. This will allow the researchers to understand the unique effect social isolation and coping efficacy have on social well-being.

Scan-related anxiety, or ‘scanxiety’, is a fear that one’s scan will indicate that current treatments have failed, as reflected by the disease returning, they may have to switch or add treatments, as well as the fear of death (Portman, 2018). A recent scoping review of scanxiety pointed to work that found patients who reported greater isolation reported higher distress on the day of their imaging (Derry-Vick et al., 2023). In the current study, researchers investigated the unique influence of interpersonal contexts (i.e., social isolation & coping efficacy) on social well-being for patients approaching lung cancer scans, and account for intrapersonal behaviors of proactive coping and scanxiety as they have been shown to be related to other internal factors that may impact social well-being.

## Present study

The present study tested whether social isolation and coping efficacy are each related to social well-being, in the months prior to a CT scan for patients with lung cancer. Researchers also investigated whether these relationships are robust by controlling for internal mechanisms of proactive coping and scanxiety, as these have been shown to be related to well-being and social support (Derry-Vick et al., 2023; Vaculíková et al., 2019). Previous work has shown that coping efficacy and social isolation are each uniquely related to social well-being for patients with cancer (Chambers et al., 2012; Gonzalez et al., 2012; Hamann et al., 2021; Hathaway et al., 2022; Luszczynska et al., 2013; Polański et al., 2022; Rigney et al., 2021; Takemura et al., 2022; Williamson et al., 2020). The current study extends this work by suggesting that coping efficacy and social well-being may operate on different timescales by considering the importance of context when looking at social well-being; namely, a patient’s upcoming CT scan. Researchers developed cross-sectional and longitudinal hypotheses based on previous work. Cross-sectionally, researchers hypothesized that higher social isolation would be related to lower social well-being. Longitudinally, researchers hypothesized that increases in coping efficacy would also be related to increases in social well-being. Researchers tested these relationships above and beyond proactive coping and scan-related anxiety.

### Methods

## Participants and procedures (screening and consent)

The present project is part of a larger study (NCATS; Grant Number: 2KR1382102) focused on identifying how patients cope with repeated scans and scanxiety. We previously published information on daily fluctuations of scanxiety and anticipatory coping in the time surrounding scans (Dunsmore & Neupert, 2023). Here, we focus on interpersonal dynamics of lung cancer-related social isolation. The research team recruited 25 patients from a North Carolina non-profit organization who were eligible if they had lung cancer and were treated with curative intent, and who were within 6 months (mode = 2 months until scan) of their upcoming CT scan. A priori power analysis (Faul et al., 2007) was conducted where researchers set the minimum threshold of desired power at 0.80 and assumed a relatively small effect size (*d* = 0.25) based on previous literature (Iida et al., 2017). Researchers assumed the correlation between repeated measures was 0.50. Results indicated that 24 participants would be needed to achieve a power level of at least 0.80. Demographics and clinical information were collected at baseline, with participants being mostly White (80%; Black: 12%), women (96%), and were between 43 and 78 years old (*M* = 62.33, *SD* = 8.10). Inclusion criteria, consent procedure, Institutional Review Board approval, as well as monthly and baseline survey procedures are described in previous work (Dunsmore & Neupert, 2023). The response rate of the possible 59 monthly surveys was 100% (mode = 2 monthly surveys per participant). All measured variables (demographics, social isolation, coping efficacy, proactive coping, scanxiety, and social well-being) were self-reported. Participants were compensated $75 for their participation in the monthly and daily parts of the original study (Dunsmore & Neupert, 2023).

## Measures

### Lung cancer stigma—social isolation (*baseline predictor*)

The shortened Lung Cancer Stigma scale assesses patient-perceived lung cancer stigma and was validated cross-sectionally (Carter-Harris et al., 2014; Cataldo et al., 2011). Participants are asked to imagine how they would feel under the circumstances described, even if they had not actually experienced that situation. The current study focused on the s*ocial isolation* subscale that includes 9 items such as “Some people who know have grown more distant”. These items were answered on a 1 (strongly disagree) to 4 (strongly agree) Likert scale. Mean scores were calculated for each person with higher scores reflecting more social isolation (α = 0.89).

## Coping efficacy—getting support (*monthly predictor*)

The shortened 13-item Coping Self-Efficacy scale (Chesney et al., 2006) is used to understand perceived coping efficacy. Participants are asked how confident they are to do the following items when things are not going well for them, using a Likert scale ranging from 0 (cannot do at all) to 5 (moderately certain can do) and up to 10 (certain can do). The current study used the three items in the ‘getting support’ subscale related to getting support from friends and family (e.g., “Get friends to help you with the things you need”, “Get emotional support from friends and family”, and “Make new friends”). Monthly mean scores were computed for the subscale; higher scores signify higher levels of coping efficacy (α = 0.72).

## Proactive coping (*monthly covariate*)

The 6-item Proactivity Scale (Aspinwall et al., 2005), was used to assess individuals’ “preference for planning for adverse events and expending resources to prevent them or to reduce their impact” [p. 365]. Participants answered items such as “I prepare for adverse events” on a 5-point Likert scale from 1 (strongly disagree) to 5 (strongly agree). Mean scores were calculated for each participant at each timepoint across items for the scale, with higher scores signifying higher levels of proactive coping (α = 0.90).

## Scanxiety (*monthly covariate*)

The Impact of Event Scale (IES-6) as modified by Bauml et al., (Sundin et al., 2002; Bauml et al., 2016), for lung cancer scan screenings, examined monthly scanxiety. It was modified for this study with a cognitive reset to reflect the last 30 days, and participants were asked to think about how distressing each difficulty had been for the past 30 days with respect to their scan from 0 (not at all) to 4 (extremely). Mean scores were calculated for each participant at each timepoint across items for the scale, with higher scores signifying higher levels of scanxiety (α = 0.85).

## Lung cancer-related quality of life—Social well-being (*outcome*)

The 44-item Functional Assessment of Cancer Therapy—Lung (FACT-L) measure assesses lung cancer-related quality of life (Cella et al., 1995). Participants are prompted with symptoms that other people with their illness have said are important and are asked to indicate their response reflecting the last 30 days. The current study only used the Social/Family well-being subscale (7 items—“I get support from my friends”). These items are scored on a 0 (not at all) to 4 (very much) Likert scale. A monthly mean score was computed, with higher scores signifying higher levels of social well-being (α = 0.82).

## Analytic plan

Multilevel modeling (MLM) was used to analyze repeated assessments (Level 1) within people (Level 2), focusing on changes in coping efficacy and social well-being on a monthly basis (Raudenbush et al., 2002). Scanxiety and proactive coping were included as covariates to elucidate the intrapersonal factors that impact social well-being and focus primarily on the interpersonal factors: social isolation and coping efficacy. Age was also included as a covariate, as we had a wide age range of individuals and could investigate the impact of age even in a small sample. Multilevel modeling allows all available data to be used from each participant, regardless of their completion rate (Raudenbush et al., 2002). Further information on MLM can be found in Electronic Supplementary Material 1. The model conducted for the current study used coping efficacy as a predictor at Level 1, social isolation as a predictor at Level 2, and proactive coping and scanxiety as covariates at Level 1, to predict social well-being.

## Results

Descriptive statistics and between-person correlations among the study variables are presented in Table I. People with better social well-being tended to report higher coping efficacy and lower social isolation. Of note, the predictors (coping efficacy and social isolation) as well as the covariates (proactive coping and scanxiety) were not significantly correlated (see Table I).

**Table I:** Descriptive statistics and intercorrelations of all study variables (N = 25)

		*M(SD)*	N(%)	1	2	3	4	5
Between-person	History of smoking (Yes)		6^a^(25%)					
	Scan History (≥ 10)		14^a^ (60%)					
	Time Since Diagnosis (Years)	6.14^b^ (5.68)						
	1. Age	62.33(8.10)		−	−	−	−	−
	2. Social isolation	2.18(0.72)		− .38	−	−	−	−
Monthly	3. Coping efficacy	7.61(1.96)		.20	− .32	−	−	−
	4. Social well-being	3.76(0.80)		.31	− .54**	.49*	−	−
	5. Proactive coping	4.08(0.39)		− .07	.18	.13	− .26	−
	6. Scan-related anxiety	2.87(1.04)		− .23	.12	− .24	− .03	.03

*p < .05; **p < .01. LC = Lung cancer. Averages and correlations are between-person. ^a^1 participant did not respond to the item, so proportion is out of 24 respondents. ^b^Average is out of 21 participants, as 4 did not provide diagnosis date

## Unconditional models

Unconditional models (models without predictors) were conducted to confirm sufficient between- and within-person variance in social well-being and coping efficacy (Raudenbush et al., 2002; Neupert et al., 2006). Results showed that 52% of the variability in coping efficacy was within-person (σ^2^ = 2.72, z = 4.20, *p* < 0.0001) and 48% was between-person (τ_00_ = 2.56, z = 2.17, *p* = 0.02). The results for social well-being showed significant variability at both levels (see Model 1, Table II). Unconditional models were also run on the Level 1 covariates; their outcomes can be seen in Electronic Supplementary Material 1.

**Table II:** Unstandardized coefficients (standard errors) of multilevel models^a^

Effect	Parameter	Model 1^a^	Model 2^b^
Monthly social well-being, β_0_			
Intercept	γ_00_	3.76***(.17)	3.73***(.14)
Social Isolation	γ_01_		-0.63**(.21)
Age	γ_02_		0.01(.02)
Coping efficacy slope, β_1_			
Coping efficacy	γ_10_		0.07*(.03)
Proactive coping slope, β_2_			
Proactive coping	γ_20_		0.01(.14)
Scan-related anxiety slope, β_3_			
Scan-related anxiety	γ_30_		0.07(.10)
Random Effects			
Variance components			
Between-person differences (τ_00_)		0.61***(.19)	0.45**(.15)
Within-person fluctuation (σ^2^)		0.08***(.02)	0.07***(.02)
*R*^2^ Within-person		11%	8%
*R*^2^ Between-person		89%	27%

*p < .05; **p < .01; ***p < .001. ^a^Model 1 refers to the results from the fully unconditional multilevel model. ^b^Model 2 refers to the results testing the main hypotheses

## Coping efficacy and social isolation predict social well-being

Results from the conditional model showed that in months prior to one’s scan when coping efficacy increased, patients reported increases in social well-being as well (see Table II, Model 2). In addition, people who reported elevated levels of social isolation at baseline reported low levels of social well-being. Proactive coping and scanxiety were not significantly related to social well-being (Table II, Model 2). When we analyzed our results among women only, we saw that these patterns were still present.^1^

## Discussion

The goal of the current study was to test whether a contributing component of health-related stigma, social isolation, and coping efficacy were related to social well-being in the months prior to one’s CT scan, among those with lung cancer. Following the transactional model of stress and coping (Lazarus et al., 1984) and the Conceptual Model of Lung Cancer Stigma (Hamann et al., 2014), researchers focused on how social isolation, as a proxy of perceived/felt stigma based on the devaluation of others, and personal attributes of coping, specifically coping efficacy, impacted social well-being in the time surrounding one’s scan. The current results show that coping efficacy and social isolation have strong impacts on social well-being, beyond other intrapersonal thought processes (i.e., proactive coping and scanxiety). This is especially true in the context of scans, as the saturation of responses in the present 6-month window of an upcoming scan, suggests that participants are highly motivated and have more time to think about their upcoming scan and how it impacts them. This saturation suggests the importance of timing and potential implications for treatment adherence, as some work has shown that psychosocial barriers impact patients’ adherence to repeated imaging (Bostock et al., 2021; Cavers et al., 2022; Young et al., 2021).

## Coping efficacy

Previous work has shown that increases in coping efficacy are related to increases in social well-being among those with cancer (Banik et al., 2017; Chen et al., 2018; Chirico et al., 2017), and as such, researchers hypothesized this direction in the context of a patient with lung cancer’s upcoming CT scan. This hypothesis was supported, such that on months when participants reported increases in coping efficacy, they also reported increases in social well-being. What is new from this finding though, is that the relationship had not been reflected in a shorter time scale (i.e., month-to-month) and had not taken into context the timing of certain triggering cancer-related events (i.e., repeated CT scans). The current study also focused on the “getting social support” component of coping efficacy, and its strong ties to social well-being. These results reinforce the importance of not just learning to cope with cancer, but also teaching patients confidence in getting support from their social networks during the time before their scan, to help improve their quality of life as it relates to social connectedness.

## Social isolation

In line with expectations, social isolation at baseline was related to low overall social well-being for individuals approaching their CT scans. While past work has shown this negative relationship cross-sectionally and across longer periods of time (Chambers et al., 2012), the current study looked at a truncated period and within an important context (i.e., repeated CT scans). Past work has shown that patients with lung cancer report experiencing the most severe stigma as compared to other cancer types (Marlow et al., 2015). The current study found that perceived/felt stigma, in particular social isolation, can have detrimental effects on one’s feeling of social connectedness and well-being as they approach a common cancer trigger, CT scans.

## Limitations and future directions

While our study has provided rich information to understanding social isolation and coping efficacy in the time surrounding one’s scan, there are some limitations. The limited sample size and homogenous dispersion (i.e., majority White, women) of the sample, though intentional for our study, should be acknowledged for future generalizability. Our work aimed to establish the precedent that coping efficacy and social well-being fluctuate around a cancer-specific event, but larger studies should intentionally recruit a more diverse sample by gender, race, and disease type or stage. In addition, the small sample size inhibited work examining between-person analyses and interactions with our other variables (i.e., clinical information, demographics), but this was outside the scope of the current project. Anti-anxiety medication or psychological treatment history was also not captured in the current study; future work should take these factors into account as previous treatment for anxiety and learned coping skills can impact how one relies on social support and interprets stigma.

In addition, we purposefully limited the clinical experiences in recruitment to patients who received curative intent treatment and who had an upcoming scan at any point in six months. To guide future intervention work on when interventions should be implemented, future studies should control the timing of recruitment to reduce bias in participation and guide implementation. Social isolation was also measured at one point in time, and future work should work to validate measures that capture fluctuating reports of social isolation, a key component of stigma, as patients with lung cancer undergo scans for the rest of their lives, and long-term survivorship and cancer stage would be important to investigate (Luszczynska et al., 2013). Finally, as our design did not allow for a causal relationship, we suggest future research test whether the relationship between coping efficacy and social well-being is causal by designing an experimental study that targets coping efficacy.

## Conclusion

This study was the first to show that both social isolation and coping efficacy were related to social well-being among those with lung cancer who are approaching their upcoming CT scans. These results suggest that social interventions focused on reducing loneliness and getting support, may be effective strategies to improve social well-being among those with lung cancer may be beneficial regardless of social isolation or other thought processes (i.e., proactive coping and scanxiety). This work also suggests that future work is needed to identify the timing for such an intervention, as scans are a particularly stressful time for patients, and improving social connectedness is especially important among those with a stigmatized cancer, like lung cancer.

### Supplementary Information

Below is the link to the electronic supplementary material.

Supplementary file1 (DOCX 16 KB)

## Data Availability

Data available upon request.

## Footnotes

1 Results women-only: [Cross-Sectional] High social isolation was related to high social well-being (γ_01_ = -0.57, t = -2.45, *p* = .02); [Longitudinal] On months with high coping efficacy, women reported high social well-being (γ_10_ = 0.06, t = 2.18, *p* = .04); in the time before their scan. Proactive coping (γ_20_ = 0.02, t = 0.12, *p* = .91) and Scanxiety (γ_30_ = 0.07, t = 0.68, *p* = .67) were not related to social well-being.
