# Supplementary material for: Social isolation, coping efficacy, and social well-being over time in patients with lung cancer
Source: J Behav Med. 2024 Jul 29;47(5):927–34. doi: 10.1007/s10865-024-00508-z (PMC11364695; doi:10.1007/s10865-024-00508-z)
Supplement: Supplementary file 1 — Supplementary file1 (DOCX 16 KB) [file 10865_2024_508_MOESM1_ESM.docx]

**Article Title: Social Isolation, Coping Efficacy, and Social Well-Being over Time in Patients with Lung Cancer**

**Journal Name: Journal of Behavioral Medicine**

**Author Name(s): Victoria J. Dunsmore, PhD*; Shevaun D. Neupert, PhD**

**Affiliation & Email (Corresponding Author): UNC Chapel Hill; vdunsmo@unc.edu**

**Justification for Multilevel Modeling**

In the multilevel modeling framework, individual change is represented through a 2-level hierarchical model (Hawkins et al., 2001). At Level 1, each person’s variability (e.g., change in social well-being levels and coping efficacy) is represented by an intercept and slope that become the outcome variables in a Level 2 model in which they may depend on person-level characteristics (e.g., social isolation) (Hawkins et al., 2001). By examining the rate of change in outcomes across multiple waves of data (e.g., every 30 days for up to 6 months before a lung cancer CT scan), multilevel modeling is a powerful and flexible approach compared to techniques that treat change in two-wave segments (Schulenberg et al., 2001). Multilevel modeling is frequently used to model intraindividual variability; that is, people’s variability around their own average. Because estimates of both between-person effects and within-person variability are possible with multilevel models (Lee et al., 1989), conclusions regarding the variability within people across occasions and the differences between people can be made. Additionally, multilevel modeling uses all available data from each participant to estimate a trajectory for that participant, controlling for the timing of that individual’s measurements (Karney et al., 1997).

**Reporting MLM Findings**

Level 1: Social Well-Being_it_ = β_0it_ + β_1it_(Coping Efficacy) + β_2it_(Proactive Coping) +β_3it_(Scan-related Anxiety) + r_it_

Level 2: β_0i_ = γ_00_ + γ_01_(Social Isolation) + u_0i_

β_1i_ = γ_10_

β_2i_ = γ_20_

β_3i_ = γ_30_

Similar to a regression analysis, the Greek letters represent the values of coefficients related to the target variables (e.g., the intercept and slope). In Level 1, the intercept, β_0it_, is defined as the expected level of social well-being for patient i. The coping efficacy slope, β_1i_ , is the expected change in social well-being uniquely associated with coping efficacy. The covariate slopes, proactive coping and scan-related anxiety, β_2i_ , β_3i_ , are the expected changes in social well-being uniquely associated with proactive coping and scan-related anxiety, respectively. The error term, r_it_, represents a unique effect associated with person i (i.e., how much that individual fluctuates or varies over time). The individual intercepts (β_0i_ ) and slopes (β_1i_ , β_2i_ , β_3i_) become the outcome variables in the Level 2 equations, where the average social well-being level for the sample when coping efficacy = 0 is represented by γ_00_ and the average slopes of coping efficacy, proactive coping, and scan-related anxiety for the sample are represented by γ_10_, γ_20_, and γ_30_, respectively. The effect of social isolation is represented by γ_01_.

**ICC Results for Covariates (Proactive Coping and Scanxiety)**

Unconditional models (models without predictors) were conducted to confirm sufficient between- and within-person variance in proactive coping and scanxiety (Neupert et al., 2006; Raudenbush et al., 2002). Results showed that 51% of the variability in proactive coping and 18% of the variability in scanxiety was within-person (**Proactive Coping**: σ^2^ = 0.11, z = 4.33, *p* < .0001; **Scanxiety**: σ^2^ = 0.23, z = 4.19, *p* < .0001). 49% of the variance in proactive coping and 82% of the variance in scanxiety was between-person (**Proactive Coping**: τ_00_ = 0.11, z = 2.40, *p* = .008; **Scanxiety**: τ_00_ = 1.00, z = 3.03, *p* = .001).

**References**

Neupert, S. D., Almeida, D. M., Mroczek, D. K., & Spiro III, A. (2006). Daily stressors and memory failures in a naturalistic setting: findings from the VA Normative Aging Study. *Psychology and aging*, *21*(2), 424-429. https://doi/10.1037/0882-7974.21.2.424

Hawkins, J. D., Guo, J., Hill, K. G., Battin-Pearson, S., & Abbott, R. D. (2001). Long-term effects of the Seattle Social Development Intervention on school bonding trajectories. *Applied developmental science*, *5*(4), 225-236. https://doi.org/10.1207%2FS1532480XADS0504_04

Schulenberg, J., & Maggs, J. L. (2001). Moving targets: Modeling developmental trajectories of adolescent alcohol misuse, individual and peer risk factors, and intervention effects. *Applied Developmental Science*, *5*(4), 237-253. https://doi.org/10.1207/S1532480XADS0504_05

Lee, V. E., & Bryk, A. S. (1989). A multilevel model of the social distribution of high school achievement. *Sociology of education*, 172-192. https://doi.org/10.2307/2112866

Karney, B. R., & Bradbury, T. N. (1997). Neuroticism, marital interaction, and the trajectory of marital satisfaction. *Journal of personality and social psychology*, *72*(5), 1075-1092. https://doi/10.1037/0022-3514.72.5.1075

Raudenbush, S. W., & Bryk, A. S. (2002). *Hierarchical linear models: Applications and data analysis methods* (Vol. 1). Sage.
